# Management Status of Myocarditis-Related Sudden Cardiac Death

**DOI:** 10.31083/j.rcm2512452

**Published:** 2024-12-23

**Authors:** Ping Yan, Shujun Yang, Tong Wang

**Affiliations:** ^1^Department of General Medicine, The First Affiliated Hospital of Guangzhou Medical University, 510062 Guangzhou, Guangdong, China; ^2^Department of Emergency, The Eighth Affiliated Hospital of Sun Yat-sen University, 518033 Shenzhen, Guangdong, China

**Keywords:** myocarditis, sudden cardiac death, management status, risk stratification, diagnostic methods, preventive measures

## Abstract

Myocarditis, a life-threatening disease that can result in cardiac arrest and sudden cardiac death, has garnered significant attention in recent years. This review provides a comprehensive overview of the management of myocarditis-related sudden cardiac death, encompassing its pathology, diagnostic methods, therapeutic strategies, preventive measures, prognostic factors, and risk stratification. Additionally, the review highlights current challenges and future directions in this field. The aim is to enhance understanding of myocarditis-related sudden cardiac death and inform clinical practice, ultimately leading to improved patient outcomes.

## 1. Introduction

Myocarditis is an inflammatory disease of the myocardium characterized by inflammatory infiltration and death of cardiomyocytes that can be caused by infection, exposure to harmful substances (such as antibiotics, anti-tumor drugs), or an overactive immune system [1, 2]. The clinical manifestations and outcomes of myocarditis vary, ranging from uncomplicated chest pain to* de novo* or worsening heart failure, chronic dilated cardiomyopathy, and sudden cardiac death (SCD) [3]. In the United States, myocarditis accounts for up to 9% of SCD among cardiovascular events [4]. Since the onset of the COVID-19 pandemic, the incidence of myocarditis and cardiovascular events related to the COVID-19 vaccine has risen. A recent national study in South Korea revealed that the risk of SCD in patients with COVID-19 vaccine-related myocarditis is approximately 1.7% [5]. Moreover, myocarditis is a significant cause of cardiovascular disease and sudden cardiac death in athletes [6], underscoring its public health significance.

Despite advancements in understanding the pathophysiology of myocarditis and SCD, several challenges remain in effectively managing these conditions [7]. Firstly, there is no consensus on the diagnosis and treatment of myocarditis, leading to variations in clinical practice and patient outcomes. Secondly, more effective treatment options are needed, especially for preventing and treating heart failure and SCD associated with myocarditis. Lastly, there is an urgent need for improved risk stratification and predictive models to identify high-risk individuals and implement personalized preventive strategies.

**Fig. 1.**
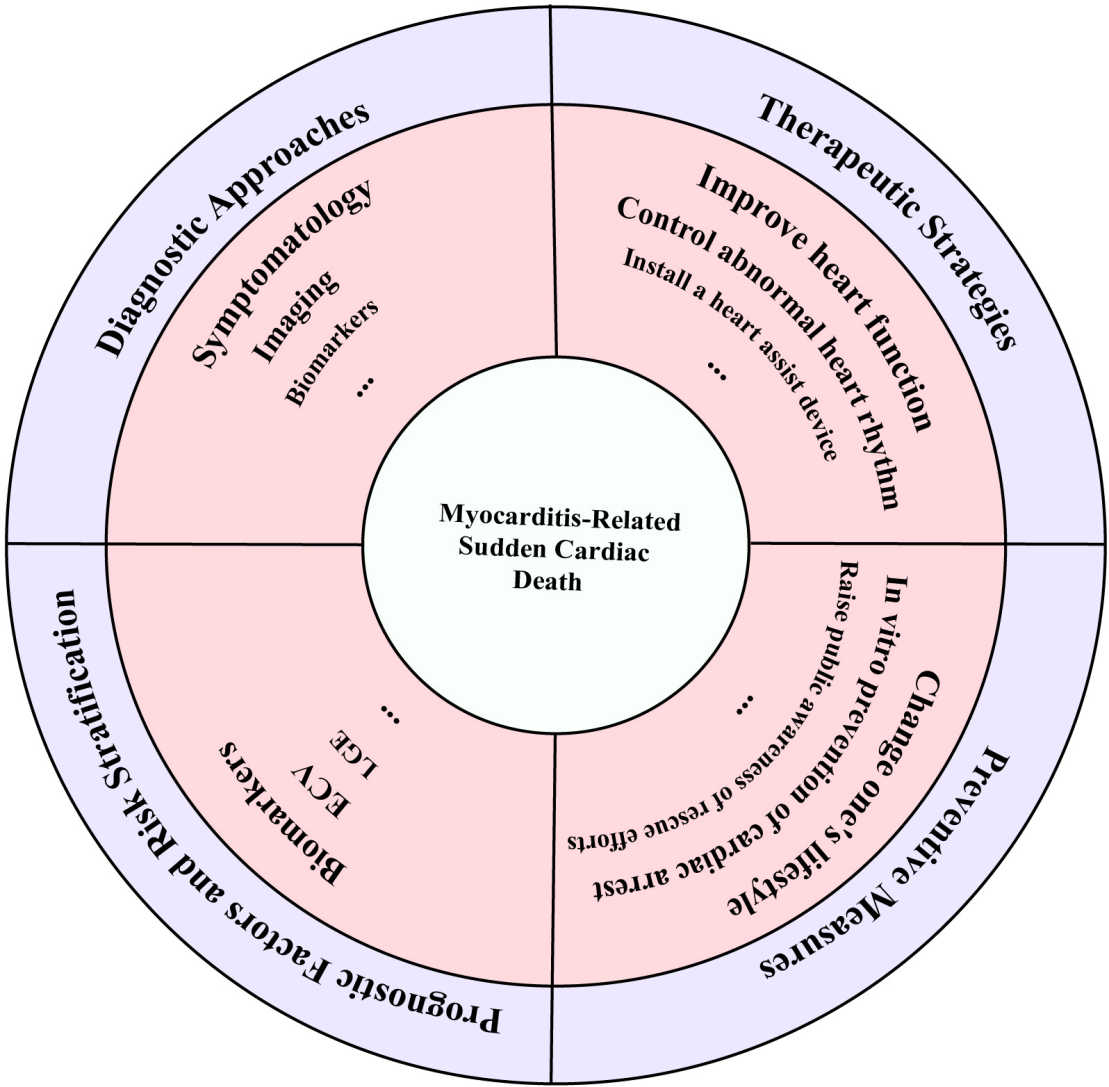
**Myocarditis-related sudden cardiac death: Summary diagram of diagnostic approaches, therapeutic strategies, preventive measures, prognostic factors, and risk stratification**.

Our report extensively references the latest research on the pathophysiological mechanisms, diagnostic methods, and treatment strategies for myocarditis. Additionally, this article delves into the evaluation methods and risk stratification for SCD and provides a comprehensive review of the current state of myocarditis management, including preventive measures and treatment options (Fig. 1).

## 2. Overview of Myocarditis Pathology

Myocarditis is inflammation of the heart that can be caused by infectious or non-infectious factors, primarily mediated by immune-induced myocardial injury. This condition is characterized by the release of pro-inflammatory mediators, leading to myocardial inflammation [8, 9]. The activation of immune cells, potentially due to specific autoantigen mechanisms, exacerbates inflammation, causing pathological remodeling and functional disruption of the myocardium [10]. Genetic variations linked to dilated or arrhythmogenic cardiomyopathy have been identified in 8% to 16% of individuals with myocarditis [11]. Patients with these pathogenic genes, particularly those affecting the structural domain of the cardiac bridge, such as the thrombophilic gene in desmosomes and the titin gene in myofibrils, often have poorer prognoses compared to those with myocarditis of other etiologies [12]. Autoantigen-reactive T cells targeting heart-specific antigens may also contribute to the pathogenesis of inflammation-related myocarditis [13].

Additionally, myocarditis can be triggered by allergies or drug toxicity. Various drugs, including antiepileptic, mood stabilizers, and diuretics, can induce myocarditis. For instance, clozapine-induced cardiomyopathy is a common clinical occurrence [14]. Eosinophilic myocarditis, caused by allergies or other factors, is often accompanied by eosinophilic infiltration, which can degranulate and release toxic cationic proteins leading to necrosis and apoptosis [15]. Lymphocytic myocarditis is a highly heterogeneous disease characterized by diffuse lymphocyte infiltration under cardioscopy [16, 17].

## 3. Diagnostic Approaches 

Over recent years, diagnosing myocarditis has been complicated by the lack of standardized methods. Endocardial myocardial biopsy (EMB) is considered the gold standard for diagnosis, and using electroanatomical mapping to guide EMB biopsies can reduce the risk of sampling errors [18, 19, 20]. A recent study have shown that EMB can predict adverse cardiovascular events in some patients with cardiomyopathy [21]. However, acute myocarditis may not be associated with clear electroanatomical abnormalities [22]. Furthermore, EMBs are often underutilized in clinical practice due to their invasive nature [2], an issue exacerbated during the COVID-19 pandemic [23]. Consequently, clinical assessments often rely on a combination of clinical symptoms, non-invasive biomarkers, and imaging characteristics [24]. Here, we conduct a comprehensive investigation into various diagnostic techniques used to evaluate myocarditis at its onset.

### 3.1 Clinical Presentation and Symptomatology

Patients with myocarditis typically present to the emergency room with symptoms such as chest pain, difficulty breathing, fatigue, heart palpitations, or fainting [25]. Research indicates that chest discomfort is the most common symptom followed by breathlessness and syncope [26, 27]. Moreover, fever is a prevalent pre-onset symptom in approximately 65% of cases. Other pre-onset symptoms, such as flu-like symptoms, gastrointestinal issues, sore throat, or upper respiratory infections, may appear days to weeks before the acute phase, with occurrence rates ranging from 18% to 80% [26, 27, 28].

### 3.2 Cardiac Biomarkers

Laboratory indicators are crucial in diagnosing myocarditis but should be interpreted alongside medical history, symptoms, and physical examination findings. Inflammatory markers such as white blood cells, C-reactive protein (CRP), and erythrocyte sedimentation rate (ESR) typically increase due to the body’s inflammatory response [26, 28]. However, these markers have low specificity and can be elevated in various other conditions, making them insufficient for a definitive diagnosis of myocarditis [29, 30].

Markers of myocardial injury, such as aspartate aminotransferase, lactate dehydrogenase, creatine kinase myocardial-bound enzyme (CK-MB), and cardiac troponin (T or I), increase in response to myocardial damage [31, 32]. Compared to CK-MB, cardiac troponin has higher sensitivity and specificity in diagnosing acute myocardial infarction [33]. Cardiac troponin reflects myocardial injury and can help assess the effectiveness of treatment for myocarditis [28]. However, while cardiac troponin can indicate myocardial injury, it cannot differentiate between ischemic and inflammatory causes of myocardial cell damage. Its diagnostic sensitivity also decreases over time, particularly after 13 days from symptom onset. This means that normal troponin levels do not rule out myocarditis [34, 35]. In chronic myocarditis, a cardiac troponin level ≥0.05 ng/mL has low sensitivity (17%), comparable to patients with non-inflammatory myocarditis whose endomyocardial biopsies are negative [36]. Nonetheless, a continuous rise in troponin levels indicates ongoing myocardial damage and may reflect disease severity [35]. Additionally, pathogenic variants of desmoplakin should be vigilant in cases of recurrent myocarditis or a family history of myocarditis [37].

### 3.3 Imaging Modalities

Echocardiography plays a vital role in the diagnosis of myocarditis [38]. This examination can identify cardiac impairment resulting from myocarditis, including regional and global myocardial function loss. Moreover, in patients with myocarditis, irregular strain analysis outcomes often indicate cardiac inflammation and edema, especially in the early stages when myocardial contraction strength is not significantly affected [39].

Cardiovascular Magnetic Resonance (CMR) is recognized as the gold standard for non-invasive tissue characterization and is crucial for patients suspected of having acute myocarditis [40, 41, 42]. Novel CMR tools, such as T1 and T2 mapping, offer higher sensitivity than traditional late gadolinium enhancement (LGE) magnetic resonance imaging. T1 mapping can detect and quantify small areas of fibrosis, while T2 mapping allows for the quantitative measurement of myocardial edema, distinguishing between acute and chronic pathologies [40]. During the disease course, detecting CMR changes within 7–14 days is optimal for observing myocardial edema or LGE. Although LGE alone has a sensitivity of about 50–60% in detecting myocarditis, combining it with the evaluation of cardiac morphology and function increases sensitivity to 83% [43]. This makes CMR a powerful tool for assessing patients with suspected myocarditis, providing detailed information about the heart and aiding clinicians in making more accurate diagnoses and treatment plans. However, it is important to note that CMR findings alone do not necessarily diagnose acute myocarditis due to the possibility of false positives, such as mild LGE abnormalities in athletes’ hearts [44]. Therefore, CMR results should be interpreted cautiously, considering clinical presentation, serology, and follow-up examinations to support or refute the diagnosis of acute myocarditis.

## 4. Therapeutic Strategies

The exact pathophysiology of myocarditis varies, often involving viral infections, autoimmune reactions, or genetic factors. Effective treatment is crucial for improving patient prognosis and preventing SCD.

Most cases of myocarditis resolve spontaneously. When the etiology is identified, treatment is tailored accordingly. For patients with treatable infections, anti-infective drugs are employed. In immune-mediated myocarditis, corticosteroids or other immunosuppressive agents may be beneficial [45]. A recent study suggest that selective immunosuppressive therapy could benefit patients with chronic myocarditis [8]. Patients with symptoms of left ventricular dysfunction are recommended to receive medications such as those targeting the renin-angiotensin-aldosterone system, beta-blockers, and diuretics [45, 46]. For those with severely impaired heart function, positive inotropic drugs, extracorporeal membrane oxygenation, or left ventricular assist devices can serve as a bridge to further treatment. The use of nonsteroidal anti-inflammatory drugs (NSAIDs) in myocarditis is controversial. While cardiomyopathy guidelines typically advise against NSAIDs [2], recent findings indicate that these drugs might protect the heart and potentially reduce the extent of LGE on CMR imaging [47]. Arrhythmia is a common manifestation in myocarditis, and treatment should follow standard arrhythmia guidelines. Heart function should be re-evaluated 3 to 6 months after an acute myocarditis diagnosis to determine the need for an implantable cardioverter-defibrillator (ICD) [48]. High-risk individuals, particularly athletes returning to low-intensity exercise, might benefit from wearable cardioverter-defibrillators as a temporary measure [49].

When considering ICD or cardiac resynchronization therapy (CRT) in myocarditis patients, it is important to assess their specific indications. Device implantation during the acute phase of myocarditis is generally not recommended to prevent SCD. For high-risk patients with arrhythmia and/or severe left ventricular dysfunction, wearable cardioverter-defibrillators may provide interim protection until more permanent solutions, such as device implantation, cardiac transplantation, or immunosuppressive therapy, are feasible [50]. However, ICD implantation should be considered even during the acute phase for patients experiencing persistent ventricular tachycardia or ventricular fibrillation leading to poor hemodynamic conditions [50]. In chronic myocarditis or recurrent ventricular tachycardia post-myocarditis, if amiodarone is ineffective or not tolerated, alternative strategies such as catheter ablation and ICD implantation should be considered [51].

## 5. Preventive Measures

Preventing SCD in patients with myocarditis is of vital importance for enhancing their long-term survival rate. Early detection and intervention are key components of a successful prevention strategy [1, 27]. Lifestyle modifications play a significant role in preventing SCD associated with myocarditis. This includes adopting a healthy diet, maintaining reasonable sleep patterns, and ensuring adequate rest. For patients with myocarditis, in addition to basic symptomatic treatment, it is essential to avoid even light exercise for at least three months to reduce the risk of SCD [52].

For individuals at high risk of sudden cardiac arrest, external defibrillators are considered a feasible prevention option. These devices continuously monitor a patient’s heart rate and can deliver immediate compressive cardiopulmonary resuscitation (CPR) upon detecting cardiac arrest, restoring blood flow and preventing the harmful effects of sudden cardiac arrest. Additionally, if the patient is conscious and presses the button, the shock process can be terminated, further ensuring safety [53].

The use of automatic external defibrillator (AED) and the performance of CPR by bystanders significantly improve neurological function and survival rates in SCD patients [54, 55]. However, regional disparities in the availability and use of these practices exist, highlighting the need for enhanced community education on AED usage and basic life support [56, 57, 58]. Studies have shown that using mobile applications to connect well-trained volunteers for emergency response can effectively reduce compression times, leading to better outcomes for SCD patients [59, 60]. Public education about the importance of compression and the early use of AED by both non-professional and professional rescuers is essential. This approach can significantly improve the survival rate of SCD patients and facilitate their recovery.

## 6. Prognostic Factors and Risk Stratification

Identifying high-risk populations among myocarditis patients who may develop SCD is vital for timely intervention and personalized management. Various clinical indicators, biomarkers, and imaging results have been studied as potential tools for risk assessment in myocarditis [61].

Research has shown a significant correlation between the baseline concentrations of blood lipids-such as the total cholesterol/high-density lipoprotein cholesterol ratio, high-sensitivity CRP, high-sensitivity troponin I, and N-terminal pro-B-type natriuretic peptide-and the future risk of SCD [62]. This correlation holds true even for individuals not yet diagnosed with cardiovascular disease, indicating that these biomarkers are predictive of SCD irrespective of an established cardiovascular disease [62]. Fluctuations in these biomarker levels could therefore serve as predictive factors for myocarditis-related SCD.

Current research also suggests that natriuretic peptides can partially reflect the risk of SCD in both the general population and individuals with coronary heart disease [62, 63]. However, there is insufficient evidence to suggest that brain natriuretic peptide can be used as a marker to assess the risk of SCD specifically in patients with myocarditis [62].

Eichhorn C *et al*. [41] highlighted that the strong correlation between CMR outcomes and patient prognosis, suggesting its potential as a central tool in risk assessment for suspected myocarditis patients. In recent years, utilizing CMR’s T1-weighted imaging technique and calculating extracellular volume fraction (ECV) significantly improves the accuracy of diagnosing myocarditis and holds great significance for assessing the prognosis of patients who present with late LGE-negative but clinically suspected myocarditis [64].

Evaluating LGE through visual assessment or semiquantitative methods can also improve the accuracy of prognosis assessment. Although the predictive value of this analysis is somewhat limited (risk ratio of 1.05 with a 95% confidence interval ranging from 1.02 to 1.08 and a *p*-value of 0.001), it remains an important observation [65]. Determining myocardial T1 and T2 values can also be used to assess myocarditis healing without the need for contrast agents [66].

Despite not being comprehensive, research indicates a relationship between LGE and the risk of cardiac death and all-cause mortality (including SCD), making CMR a crucial tool for assessing acute myocarditis [42, 43, 67]. Isolated left ventricular ejection fraction (LVEF) damage significantly increases the mortality rate. Even with normal left ventricular function, a positive LGE indicates an increased risk of major adverse cardiovascular events, including death, heart failure decompensation, heart transplantation, persistent ventricular arrhythmia lasting more than 30 seconds, and recurrent acute myocarditis [42]. The negative predictive value of CMR, especially when combined with LGE, is clinically significant as patients with biopsy-documented myocarditis who exhibit normal CMR findings have a favorable outcome [43, 68].

Re-evaluation of LGE after six months can be helpful for risk assessment. During the six-month CMR, patients with no edema and LGE had a poorer prognosis, particularly when this pattern was present in the mid-myocardial interval. The absence of edema in LGE could indicate a clear diagnosis of fibrosis, while the presence of edema suggest there is still a chance for recovery [69].

## 7. Current Challenges and Future Directions

In recent years, there has been growing recognition of the serious health risks associated with myocarditis, which can lead to severe cardiovascular events, including SCD, if left undiagnosed or untreated [70]. Despite advances, current diagnostic and therapeutic strategies for myocarditis still face several challenges.

A major limitation of current diagnostic tools is their lack of specificity and sensitivity. The nvasive nature of surgical procedures may pose significant risks to patients, limiting their use in certain clinical settings. Traditional imaging techniques, such as echocardiography, often fall short in terms of resolution and ability to accurately detect myocarditis or its underlying causes. Although CMR has gained prominence for diagnosing myocarditis and assessing SCD risk, there remains a lack of large-scale, multi-center clinical studies to further validate its effectiveness.

In terms of treatment, current strategies primarily rely on empirical evidence and expert opinions, with limited support from randomized controlled trials. This has led to considerable variability in treatment practices across different centers and countries. Personalized medicine, which considers individual genetic profiles, medical history, and lifestyle factors, holds promise for improving patient outcomes. However, the application of genomics in clinical practice is slow due to the complexity of disease pathogenesis and the rarity of some conditions.

The discovery of biomarkers has the potential to significantly advance the diagnosis and management of myocarditis. Cardiac troponin and other molecules are specifically elevated in patients with myocarditis and can aid in early detection and prognosis [71]. Advances in high-throughput sequencing and bioinformatics have revealed several potential biomarkers, though their clinical utility is still being established [72].

Artificial intelligence (AI) and machine learning (ML) offer transformative potential in medicine by processing vast amounts of data, recognizing intricate patterns, and making highly accurate predictions [73]. In myocarditis, AI and ML could automate image analysis, predict disease progression, and personalize treatment strategies based on individual genomic profiles [74].

Future research is likely to be shaped by advances in genomics, bioinformatics, and AI/ML. The development of more accurate diagnostic tools, refinement of personalized treatment strategies, and the integration of novel therapeutic approaches such as stem cell therapy are anticipated. Further exploration of the interaction between host factors and the immune responses in myocarditis will be crucial for understanding this complex condition.

In conclusion, while significant progress has been made in understanding and managing myocarditis, substantial work remains. Continued development of new diagnostic tools, establishment of personalized treatment protocols, and exploration of cutting-edge therapies are critical for improving patient outcomes. The application of AI and ML in medicine holds immense potential for revolutionizing the diagnosis and treatment of myocarditis and related cardiovascular diseases.

## 8. Conclusions

Currently, limitations persist in the diagnosis and treatment of myocarditis. Future research should concentrate on enhancing diagnostic tools and developing personalized treatment strategies, as well as investigating novel therapeutic approaches. These efforts are crucial for mitigating the impact of myocarditis and SCD.
